# Two Unique Cases of Renal Hemorrhage*Read before Brazos Valley Medical Society at Cameron, Texas, November 13 and 14, 1900.

**Published:** 1901-04

**Authors:** J. P. Oliver

**Affiliations:** Caldwell, Texas


### TEXAS MEDICAL JOURNAL.

ESTABLISHED JULY, 1885.

PUBLISHED MONTHLY.—SUBSCRIPTION $1.00 A YEAR.

VOL. XVI. AUSTIN, APRIL, 1901. No. 10.

### Original Contributions.

For Texas Medical Journal.

### Two Unique Cases of Renal Hemorrhage.*

J. P. OLIVER, M. D., CALDWELL, TEXAS.

On May the 4th I was called in haste to see Lucy, a negress, about twenty-two or three years of age, multipara, seven months advanced in gestation, and obtained the following history of her case: In the forenoon she had been out in the woods gathering dewberries, and to use her exact words "saw a snake and became badly scared." In a very sort time she began to sicken and to have some pains in the lower part of the abdominal region. As soon as she could do so she returned home and went to bed. After several hours of pain and vomiting she sent for me, detailing the above history of her case. My first impression was that the fright, if any, was going to produce premature labor, as from the indications and symptoms she seemed to be in labor. A vaginal examination was had and negative signs of labor revealed.

I felt somewhat puzzled over my first impression of her case, and felt quite at a loss for several minutes to account for the pains she was having. But as she was over the chamber every few minutes, and my vaginal examination had revealed negative results, it occurred to me that the pains possibly might be vesical, so I introduced my catheter and, to my surprise, drew off a considerable

*Read before Brazos Valley Medical Society at Cameron, Texas, November 13 and 14, 1900.

amount of bloody urine. If I recollect aright, there was very little rise of temperature, not over 100, but the circulation was quite feeble and depressed, respirations quickened, general indications of prostration. Thinking that I might have a case of malarial hamaturia developing, after administering an anodine and arterial stimulants, I gave her four portions of calomel and soda 2½ grains each, also a dieuretic of bicarb. potash and ordered her sister to let let me know the results, still believing that labor would supervene before the trouble would terminate. This hemorrhage continued four days under the above treatment, save some quinine to control temperature when it ceased and my patient made an uneventful recovery, and went on to her full time of delivery, with no rise of temperature above 100.

The second case was a little white girl between 6 and 8 years of age, who was bitten or supposed to have been bitten by a small spider Sunday morning, May 13, 1900, over the right shoulder blade. I believe she was up all day, and did not complain but very little until about sundown. I was sent for in great haste, as the little girl was supposed to be almost in articulo mortis. On my arrival the above history was related to me by her cousin. The little patient was in a very critical condition, as was manifested by weak circulation, some delirious condition and general prostration. I administered 1-50th grain of sulphate of strychnia and 1-100th grain of nitro glycerine hypodermatically and gave some aromatic spirits of ammonia by the mouth and a small dose of morphia and atropia, 1-16th gr. of the former and 1-50th gr. of the latter, to allay pain and produce quietude of the nervous system, as she seemed to be in great agony. In the course of one or two hours there was considerable reaction and I went home, with instructions that if she grew worse to let me know, and to continue the ammonia and alcoholic stimulants all night at such intervals as her condition demanded. Very early next morning a message was sent me that the little girl was worse, that about 12 p. m. she began to have hemorrhages from the kidneys. On examination after my arrival of the place bitten over the shoulder blade, I found an extravasation of blood all over the back and as low down as the points of the shoulder blades and down the chest to the epigastric region, an icteroid condition over the balance of the surface appeared. About this time my friend, Dr. Kruegar, came in to see one of the same family. I requested him to see the little girl with me which he readily consented to do. This little girl's temperature never rose above one hundred, if I mistake not. But the most prominent symptoms were great nervous prostration, weak circulation and a continuation of the delirious state. We opened the bowels, kept up the heart stimulants every two or three hours, gave liquid nourishments as best we could. For several days the general indications were that we would lose our little patient in spite of all our efforts. I write from memory, but I think the hemorrhage continued for four or five days and then ceased. She, too, recovered but tediously. For several days we thought that the bitten part would slough, but it did not.

REMARKS.

In the case of insect bites the poison or venom inoculated is acid in nature, so the classical authorities state. The results of its injection produces every degree of variation in severity from simple local irritation to extreme prostration, and death may ensue. As a rule, however, the stings of the most venomous insects are not attendant with danger to life. The wounds inflicted by scorpions and spiders of the largest size rarely prove fatal. Naturalists tell us that there are 27 species of poisonous serpents and poisonous lizards in the United States. Eighteen species of these are true rattlesnakes, the remaining number are divided between the moccasin or copperhead and the viper. The lizard is the Texas reptile known as the Gila monster. The physical appearances of all serpent venom is nearly alike. It is a viscid fluid, varying in color from a pale amber to a deep yellow and containing in solution albuminous principles, which are the toxic elements,the nature of which has not been made out.

According to the researches of Mitchell and Reichert, venom induces rapid necrotic changes in the living tissues with which it is brought in contact. It renders the blood uncoagulable, disintegrates the red blood corpuscles and produces such a change in the capillary blood vessels that their walls are unable to resist the normal blood pressure and wide and rapid blood extravisation results. In my opinion we have in the little girl a combination of the above authors quoted. The tissues did not become necrotic, but a deep discoloration was present with considerable extravisation of blood as was evidenced by the general hemorrhagic condition as well as the hemorrhage from the kidneys.

Were these two cases simply coincident cases occurring within four or five days of each other, or were the same pathological conditions present in both? Is it not possible that the colored woman may have been bitten by some insect, or perhaps by the snake that produced the scare, in some very vascular part? That the sudden thrusting of either the venom of the sting of an insect or the bite of a serpent into her circulation was the cause of the sudden development of the hemorrhage in her case, obtains without a reasonable doubt. And the delay of the development of the hemorrhage in the little girl's case may be accounted for by the slow absorption of the venom. Would the pregnant state of the colored woman have such a reflex influence over her nervous system from the scare as to produce a paretic condition of the vaso motor system, and through that over the walls of the capillary vessels of the right side of the heart so that they could not resist the normal blood pressure and produce the hemorrhage by exosmosis or a transudation through the walls of the blood vessels?

In the little girl's case her mother in the latter part of her life had manifested symptoms of insanity. Was the cerebral condition of the mother transmitted to the child and rendered its nervous system more susceptible to any poisonous element, or was it due to the venom of the insect operating upon the nervous centres and through them upon the capillary blood vessels and producing the hemorrhagic state above described?

The post mortem appearances are such as would follow the blood and the visceral changes above described. In the neighborhood of the bite the tissues are infiltrated with hemorrhages as was in the case of the little girl. We were very apprehensive that gangrene would occur about the bitten part, which is not unfrequently the case. If death results the right side of the heart is engorged, general blood mass is fluid, and the internal organs, brain, spinal cord, and kidneys are congested and present multiple ecchymosis.

